# Comparison of Marginal Microleakage of Glass Ionomer Restorations in Primary Molars Prepared by Chemo-mechanical Caries Removal (CMCR), Erbium: Yttrium Aluminum-Garnet (Er:YAG) Laser and Atraumatic Restorative Technique (ART)

**DOI:** 10.5005/jp-journals-10005-1193

**Published:** 2013-08-26

**Authors:** Apa Juntavee, Niwut Juntavee, Jomjai Peerapattana, Nartsajee Nualkaew, Sitikorn Sutthisawat

**Affiliations:** Associate Professor, Department of Pediatric Dentistry, Faculty of Dentistry, Khon Kaen University, Thailand, e-mail: apa.edu@hotmail.com; Associate Professor, Department of Prosthodontics, Faculty of Dentistry, Khon Kaen University, Thailand; Associate Professor, Department of Pharmaceutical Technology Faculty of Pharmaceutical Sciences, Khon Kaen University, Thailand; Lecturer, Department of Pharmacognosy and Toxicology, Faculty of Pharmaceutical Sciences, Khon Kaen University, Thailand; Dental Student, Department of Pediatric Dentistry, Faculty of Dentistry, Khon Kaen University, Thailand

**Keywords:** Marginal microleakage, Glass ionomer restorations, Chemomechanical caries removal, Erbium:yttrium aluminum-garnet (Er:YAG) laser, Atraumatic restorative technique

## Abstract

**Background:** It is important to emphasize that the aspects of pretreatment techniques, as well as the composition and mechanism of adhesion, may decisively influence the effectiveness of the restorative materials in sealing cavity margins and preventing marginal leakage.

**Aims:** This study assessed the *in vitro* influence of surface preparation techniques on the microleakage of glass ionomer restorations in primary teeth.

**Materials and methods:** The study groups were divided into three different techniques: (1) The chemomechanical caries removal (CMCR) method using the Apacaries gel, (2) the erbium:yttrium aluminum-garnet (Er:YAG) laser method and (3) the atraumatic restorative technique (ART). The teeth restored with a glass ionomer restorative material (Fuji IX GP capsule, GC Corporation, Tokyo, Japan). The dye penetration was measured in micrometers using a polarized light microscope and specific computer software**.**

**Results**: The results showed that the mean microleakage level after was lowest with the CMCR method using Apacaries gel and highest with the Er:YAG laser. There was a statistically significant difference regarding the mean microleakage level between the group with the CMCR method using Apacaries gel and the Er:YAG laser.

**Conclusion:** Marginal leakage was significantly higher with preparations made using the Er:YAG laser than with the CMCR method using Apacaries gel and spoon excavator (p < 0.05).

**How to cite this article:** Juntavee A, Juntavee N, Peerapattana J, Nualkaew N, Sutthisawat S. Comparison of Marginal Microleakage of Glass Ionomer Restorations in Primary Molars Prepared by Chemomechanical Caries Removal (CMCR), Erbium: Yttrium Aluminum-Garnet (Er:YAG) Laser and Atraumatic Restorative Technique (ART). Int J Clin Pediatr Dent 2013;6(2):75-79.

## INTRODUCTION

In the past, most caries treatment techniques have involved hand instruments, which was painful, ineffective and tedious for caries removal.^1^ These disadvantages have led to the evolution of rotary instruments, ranging from low speed to ultra-high speed equipment. However, major drawbacks associated with rotary instruments are thermal and pressure effects on the pulp, which produce pain.^2^ The search for a more gentle, comfortable, and conservative technique for caries excavation has led to the development of methods that produce minimal thermal changes, less vibration and pain, and removal of infected dentine.^3^ Such alternative techniques include air abrasion, ultrasonic instrumentation, lasers and a chemomechanical approach to caries removal. Erbium:yttrium aluminum-garnet (Er:YAG) laser is one method of cavity preparation considered to be less traumatic because it produces minimal vibration, creates less noise, and requires no local anesthesia. These characteristics make Er:YAG laser ideal for pediatric clinical use.^4-6^ Atraumatic restorative technique (ART) is another contemporary approach that uses sharp-edged hand instruments to minimize the loss of tooth structure by removing only the infected dentin. However, it is often difficult to establish exactly how much dentin should be removed, given the lack of objective clinical markers.^7^ Furthermore, mechanical preparation often induces pain and discomfort, thereby requiring local anesthesia.^5^ Chemomechanical caries removal technique (CMCR) is an alternative approach to traditional mechanical caries removal that involves the use of a chemical product to soften the carious dentin followed by removal with gentle excavation.^8^ Apacaries gel is a novel dental material composed of polyphenol in mangosteen extracts and papain, mixed in a gel preparation; this material was developed for caries removal involving gentle excavation in primary teeth.

In very young and uncooperative children, both the ART and CMCR approaches followed by a glass ionomer restoration are considered appropriate in pediatric dental caries management. After cavity surface preparation, glass ionomers are the restorative material of choice, due to their ability to bond chemically to both dentin and enamel, as well as their biocompatibility, favorable thermal expansion and fluoride release. Therefore, glass ionomer is considered to be the ideal material for precooperative, uncooperative and/or high risk caries children. However, the adaptation of glass ionomer materials to the cavity walls after caries removal is an important factor that can lead to marginal microleakage, which is a major problem in clinical dentistry. Microleakage is defined as the clinically undetectable passage of bacteria, nutrients, fluids, molecules or ions between cavity walls and restorative materials applied to them.^9^

## AIM

The purpose of this study was to assess the *in vitro* influence of the following three cavity surface preparation techniques:

(1) CMCR using the Apacaries gel and gentle excavation,

(2) Er:YAG laser and (3) ART using a spoon excavator

## MATERIALS AND METHODS

Primary second molars that were collected from extracted deciduous teeth and stored in a thymol solution at 25°C for fewer than 6 months were used in this study. Sixty extracted primary molars with occlusal caries involving dentine were selected and randomly divided into three groups (n = 20 each) according to caries removal and preparation methods as described in Table. 1. In group 1, Apacaries gel was applied for 30 seconds onto the carious occlusal surfaces before the carious dentin was removed with a spoon excavator (Hu-Friedy, Chicago, IL, USA). In group 2, carious dentine was removed using the ART with a spoon excavator (Hu-Friedy, USA). In group 3, carious dentin was removed using the Er:YAG laser (Fotona, R02-C, Slovenia). The laser was set at 260 mJ, 30 Hz in pulse mode with the water-cooling system. Carious dentine was removed in all samples until all softened carious dentine remained. A dental explorer was used to assess the remaining hard dentin. Carious tissue removal was determined using a laser fluorescence device (Diagnodent, Kavo Dental, USA).

The cavities received an application of 10% polyacrylic acid (GC Corp., Japan) for 20 seconds before they were thoroughly rinsed with water. Glass ionomer restorative materials (Fuji IX GP, GC Corp., Japan) were used as restorative filling materials. After complete setting, the glass ionomer restorative surfaces were protected using a petroleum jelly (Vaseline, Johnson & Johnson, USA). All specimens were stored in a 100% humidifier at 37°C for 24 hours prior to completion of the restoration surface using a moist polishing set (Super-Snap disks, Shofu Inc, Kyoto, Japan). Immediately afterwards, finishing gloss (3M, USA) was applied; and the restorations were polymerized for 20 seconds. All tooth samples were subjected to thermocycling (KMITL, TC300, HWB332, CWB332, Thailand) for 500 cycles between 5 and 55°C, 30 seconds of immersion in each bath, and 30 seconds of transfer time between conditioning.

**Fig. 1:**
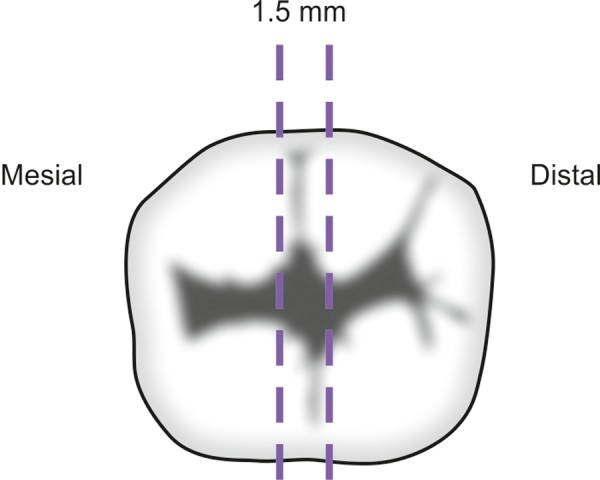
Teeth were bisected longitudinally in a buccolingual direction, providing 1.5 mm thickness cuts per tooth

To assess microleakage in the restorations, samples were dried superficially with absorbent paper and sealed with 2 coats of nail varnish, leaving a 1 mm window around the cavity restoration margins. The apical region of each tooth was also sealed with epoxy glue to prevent dye penetration. Specimens were subsequently immersed in a 2% methylene blue solution at pH 7 and 37°C for 4 hours, after which the surface-adhered dye was rinsed in tap water, and the epoxy resin and nail varnish were removed with a sharp instrument and dried with absorbent paper. The tooth samples were embedded in chemically activated acrylic resin (JET, Cléssico, São Paulo, SP, Brazil) and bisected longitudinally in a buccolingual direction with the water-cooled diamond saw of a sectioning machine (Isomet 4000^®^, Buehler, IL, USA), providing 1.5 mm thickness cuts per tooth as shown in Figure 1. Subsequently, the cuts were initially thinned in a polishing machine (Politriz, Struers A/S) using #180- to #600-grit wet silicon carbide papers before they were manually smoothed with #1000- and #1200-grit wet silicon carbide papers to obtain a flat surface and an average final thickness of 1 mm.

**Table 1:** **Table 1:** Techniques and materials used for caries removal, surface treatment of cavities and subsequent restoration

*Group*	*Cavity preparation*	*Surface treatment*	*Restoration*
1	Apacaries gel (apply for 40 seconds, followed by gentle excavation)	10% polyacrylic acid (20 seconds application before rinsing with water)	Glass ionomer (Fuji IX GP, GC Corp., Japan)
2	ART using a spoon excavator (Hu-Friedy, USA)	10% polyacrylic acid (20 seconds application and rinse with water)	Glass ionomer (Fuji IX GP, GC Corp., Japan)
3	Er:YAG laser (Fotona, R02-C, Slovenia, 260 mJ, 30 Hz)	10% polyacrylic acid (20 seconds application and rinse with water)	Glass ionomer (Fuji IX GP, GC Corp., Japan)

**Figs 2A to D:**
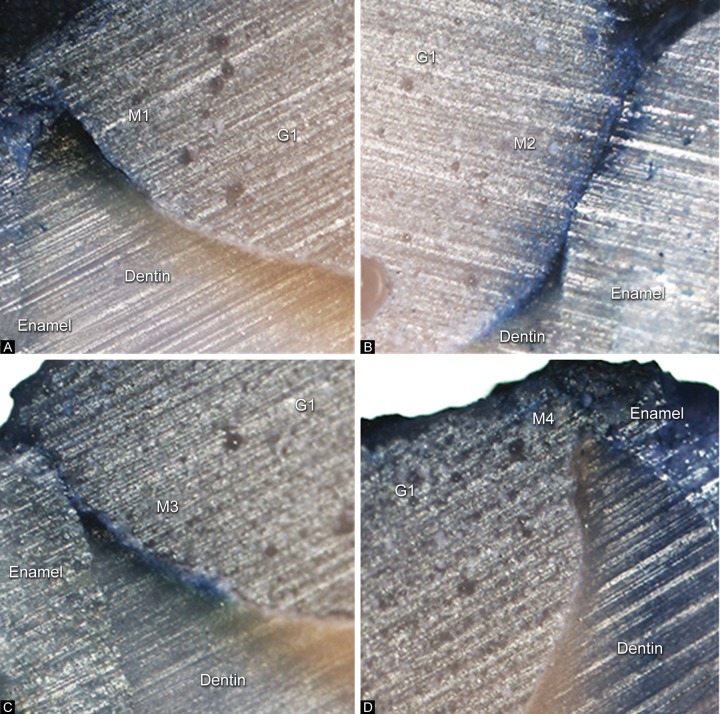
Polarized light microscopy indicated microleakage of the dye penetration on the mesial and distal sides of the specimen. (A) Length of the dye penetration on the buccal part of the mesial side; M1, (B) length of the dye penetration on the lingual part of the mesial side; M2, (C) length of the dye penetration of the buccal part of the distal side; M3 and (D) length of the dye penetration on the lingual part of the distal side; M4

The degree of dye penetration (microleakage) was examined using a polarized light microscope (Nikon Eclipse 80i, Kanagawa, Japan) and the distance of penetration was measured. Microleakage measurements along the cavity walls were obtained from four points, involving the buccal and lingual parts of both the mesial and distal sides, as shown in Figures 2A to D. The penetration distances of the dye were later determined and measured. Mean and standard deviation values were calculated for each group. Differences in microleakage values between the tested groups were then analyzed statistically using analysis of variance (ANOVA) and Scheffe's pairwise post hoc multiple comparisons.

## RESULTS

**Fig. 3:**
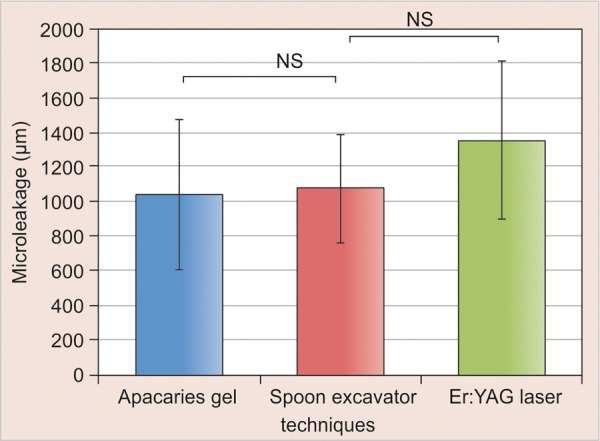
Means and standard deviations for the three study groups

The means, standard deviations and 95% confidence intervals for each group are shown in Table. 2 and Figure 3. ANOVA revealed significant differences in mean micro-leakage among the groups (p < 0.05), as shown in Table. 3, indicating significance differences in the glass ionomer restoration as a result of different carious removal methods. Scheffe's post hoc multiple comparison revealed significant differences in microleakage between cavities prepared with Er:YAG laser and Apacaries gel (p < 0.05), as shown in Table. 4. The mean microleakage of glass ionomer restorations in cavities prepared using atraumatic restorative carious dentin removal technique demonstrated higher levels of microleakage than did cavities prepared by Apacaries gel but less than those using Er:YAG laser, as shown in Figure 3 and Table. 2. However, these differences in microleakage indicated no statistically significant differences between ART and Apacaries gel for carious dentine removal technique (p > 0.05) nor was a significant difference observed between ART and Er:YAG laser (p > 0.05).

**Table 2:** **Table 2:** Means, standard deviations and 95% confidential intervals for dye penetration among the experimental groups

*Methods*	*N*	*Microleakage (μ)*			*95% confidence interval*	
		Mean	SD		Upper limit	Lower limit
Apacaries gel	20	1042.99	429.7		841.89	1244.09
Spoon excavator	20	1075.51	312.69		929.16	1221.84
Er:YAG laser	20	1356.23	458.38		1141.70	1570.76

N: Sample size; SD: Standard deviation; μ: Micron

**Table 3:** **Table 3:** ANOVA of the dye penetration for the tested groups

	*SS*		*df*		*MS*		*F*		*p-value*
Between groups	0.168		2		0.084		3.788		0.029
Within groups	1.263		57		0.022		–		–
Total	1.431		59						

SS: Sum of square; SD: Mean square; F: Degree of freedom; p: probability

**Table 4:** **Table 4:** Scheffe's post hoc multiple comparison of dye penetration for the tested groups

*Groups*	*Apacaries gel*	*Spoon excavator*	*Er:YAG laser*
Apacaries gel	1.000	0.877	0.041
Spoon excavator	–	1.000	0.123
Er:YAG laser	–	–	1.000

## DISCUSSION

The results of this study showed that mean microleakage levels associated with caries removal and cavity preparation with CMCR using Apacaries gel was lowest compared to ART and Er:YAG laser. There was a statistically significant difference between mean microleakage levels for the CMCR and Er:YAG laser groups (p = 0.041). Although the laser mechanism has not been well defined, a suitable explanation for such finding would be that the laser creates a specific cavity configuration. In addition, laser application of the dental substrate may promote the disorganized destruction of enamel prisms. It is also possible that the irregularity of the walls, internal angles and margins interfered with the interaction between the restorative material and tooth structure, thereby compromising the marginal sealing of the restorations and favoring marginal leakage.^10^ The smaller amount of dye penetration in cavities prepared by CMCR with the Apacaries gel may be attributed to the mechanical removal of the dental substance without heat generation. Therefore, this method may not have affected the composition and/or ultrastructure of either the organic or inorganic components of the tooth substrate. In addition, it should be noted that the Er:YAG laser's selective ablation of collagen-rich inter tubular dentin coupled with the photothermal effect causes the decomposition of the organic contents as well as the degradation, collapse, or even melting of the collagen fiber mesh, which obliterates the tubular openings and restricts the subsequent interdiffusion of the dentin conditioner. The disposition of the hydroxyapatite crystals in the aprismatic layer, consisting of hydroxyapatite crystals arranged parallel to each other and perpendicularly to the enamel surface, has been reported to affect the quality of the adhesion.^11,12^ The adhesion mechanism of the glass ionomer cements to Er:YAG laser-prepared tooth surfaces is not yet fully understood. Based on rather limited data,^13-20^ it can be concluded that the adhesion process can be influenced by the following several parameters: The irregular cavity outlines and crater-like character of the smear layer free surface, changes in the calcium-to-phosphorus and carbon-to-phosphorus ratios, increased acid resistance of the irradiated surface, evaporation of dentinal moisture during irradiation, formation of a subsurface layer with cracks, and glazing/ melting of superficial crystalline microstructures.^21^ It is important to emphasize that the intrinsic aspects of pretreatment techniques, as well as the composition and mechanism of adhesion, may decisively influence the effectiveness of the restorative materials in sealing cavity margins and preventing marginal leakage. Further studies should aim to understand better the structural alterations influenced by the conditioning of tooth surfaces prepared by CMCR with Apacaries gel and their effects on the adhesive mechanisms of glass ionomers to the enamel and dentine. The adhesive interface micromorphology and alterations in the substrate compounds following CMCR with Apacaries gel should also be assessed.

## CONCLUSION

Marginal leakage in primary teeth restored with glass ionomer was significantly higher in preparations using the Er:YAG laser than in preparations using CMCR with the Apacaries gel and the ART.

### What This Paper Adds?

- To the best of our knowledge, this report describes the first *in vitro* study assessing the microleakage of glass ionomer restorations after CMCR between Apcaries gel and Er:YAG laser in pediatric dental therapy.

### Why This Paper is Important for Pediatric Dentists?

- Pediatric dentists should pay attention to and seek alternative restorative treatments that deliver more comfort, efficiency and cost-effectiveness in caries management in children.
  A novel approach using a CMCR with Apcaries gel may be a valuable tool in the management of early childhood caries and of apprehensive patients.
